# SHAP-explained machine-learning model for high-risk gastric cancer identification

**DOI:** 10.3389/fonc.2026.1732072

**Published:** 2026-03-16

**Authors:** Hyun Jin Oh, Chung Ho Kim, Jae Kwan Jun, Mina Suh, Kui Son Choi, Il Ju Choi, Bomi Park

**Affiliations:** 1 Division of Gastroenterology, Department of Internal Medicine, Center for Cancer Prevention and Detection, National Cancer Center, Goyang, Republic of Korea; 2 Department of Preventive Medicine, College of Medicine, Chung-Ang University, Seoul, Republic of Korea; 3 National Cancer Control Institute, National Cancer Center, Goyang, Republic of Korea; 4 Division of Gastroenterology, Department of Internal Medicine, Center for Gastric Cancer, National Cancer Center, Goyang, Republic of Korea

**Keywords:** atrophic gastritis, gastric cancer, *Helicobacter pylori*, intestinal metaplasia, risk prediction, Shapley Additive Explanations

## Abstract

**Introduction:**

Gastric cancer (GC) remains a major public health concern in Asia. Risk prediction tailored to regional biological features such as *Helicobacter pylori* (*H. pylori*) status and high-risk mucosal findings such as atrophic gastritis (AG) and intestinal metaplasia (IM) may help improve the screening workflow.

**Methods:**

Using a large, real-world, nationwide screening cohort with available endoscopic AG/IM codes, we developed 2-year GC risk prediction models that integrate AG/IM with regional demographic and lifestyle factors. We compared a conventional Cox proportional hazards model (CPHM) with the following machine learning (ML) approaches: extreme gradient boosting (XGBoost), decision tree (DT), and logistic regression (LR). Discrimination and calibration were evaluated through internal and external validations. Model interpretability was assessed using Shapley Additive Explanations (SHAP).

**Results:**

The XGBoost model demonstrated the best overall performance, achieving an AUROC of 0.764 (95% CI, 0.755–0.767), a sensitivity of 0.607 (95% CI, 0.560–0.650), and a specificity of 0.746 (95% CI, 0.744–0.750) in the internal validation. In the external validation cohort, XGBoost also showed the highest discrimination with an AUROC of 0.708 (95% CI, 0.682–0.884), a sensitivity of 0.666 (95% CI, 0.470–0.830), and a specificity of 0.597 (95% CI, 0.590–0.600). SHAP analysis consistently identified Helicobacter pylori infection, age, sex, smoking, and atrophic gastritis/intestinal metaplasia (AG/IM) as the major contributors to increased predicted gastric cancer risk.

**Discussion:**

This externally validated and interpretable short-term GC risk model incorporating endoscopically ascertained AG/IM could provide a practical approach for informing risk-adapted screening workflows. The model could help identify individuals at a higher predicted risk for prospective evaluation and closer clinical review. In addition, SHAP clarifies the main contributors to each prediction by highlighting factors most strongly associated with a higher predicted risk.

## 1 Introduction

Gastric cancer (GC) is the fifth leading cause of cancer mortality worldwide (1). Despite the poor prognosis of advanced GC, early detection yields markedly better outcomes, with 5-year survival exceeding 90% for early GC (2, 3). Endoscopy-based screening is widely recognized as the most effective strategy to lower GC mortality through early detection (4–6).

Normal gastric mucosa can progress to cancer through sequential precancerous stages (7), including gastritis, atrophic gastritis (AG), intestinal metaplasia (IM), and dysplasia. Therefore, more proactive management and consideration of shorter endoscopic intervals for high-risk individuals with AG or IM are an important public health priority in Korea, where GC burden is high (8). Most national guidelines recommend screening for high-risk individuals (9, 10). In Korea, since 2002, the National Cancer Screening Program has provided biennial screening endoscopy for all individuals aged ≥40 years.

However, because the National Cancer Screening Program uniformly offers biennial upper endoscopy to all eligible adults, the program’s scale (~7.76 million screening endoscopies in 2021 and 7.84 million in 2022) means that publicly financed expenditures for gastric-cancer screening clearly exceed KRW 250 billion annually (11, 12). There is a growing need to tailor screening intervals according to individual GC risks to reduce unnecessary examinations and concentrate resources on high-risk groups, thereby improving efficiency.

Here, using a large-scale cohort, we aimed to develop a Korean population-specific GC risk-prediction model that is clinically implementable and highly accurate by including not only family history and lifestyle factors but also endoscopically ascertained high-risk mucosal findings, such as AG, IM, and *Helicobacter pylori* (*H. pylori*) infection status as risk factors. In contrast to most prior GC risk models that rely on conventional statistical methods, our study applied machine-learning approaches to substantially enhance predictive performance.

## 2 Materials and methods

### 2.1 Study design and participants

This study was based on the Korean National Health Information Database (NHID), which integrates nationwide claims and health screening records and covers nearly the entire Korean population through the National Health Insurance (97%) and Medical Aid (3%) (13). Using this database, we identified adults aged 40–74 years who underwent gastroscopy as part of the National Cancer Screening Program (NCSP) in 2018, with a screening participation rate of 61.9% (14). We restricted the analysis to ages 40–74 to reflect the core NCSP screening population (screening offered from ≥40 years) and to reduce heterogeneity from advanced frailty/competing risks at older ages. Individuals with a prior diagnosis of GC or those who died during the 2-year follow-up period were excluded from the study. A total of 6,863,103 individuals met inclusion criteria. Participants were followed up until 2020 to identify patients with GC within 2 years of post-screening. Incident GC was defined according to the International Classification of Diseases, 10th revision (ICD-10) code C16, as a primary or secondary diagnosis in combination with an NHIS special registration code (V193 or V194), indicating eligibility for the expanded co-payment reduction program.

Because the 2-year GC incidence was low, we addressed class imbalance only in model training by applying the random oversampling example (ROSE) procedure to obtain an approximately 1:1 ratio of GC to non-GC; the validation/test sets retained the original outcome prevalence. We selected ROSE over alternative approaches such as synthetic minority over-sampling technique (SMOTE) or class weighting because it generates synthetic samples from a smoothed bootstrap distribution, better preserving the multivariate structure of the minority class and reducing the risk of creating noisy or borderline cases (15, 16). In contrast, class weighting does not modify the empirical feature distribution, which may limit model learning in extremely sparse outcomes (17, 18). Baseline characteristics between GC and non-GC groups were compared using χ² tests, as all variables were categorical.

### 2.2 Variables and datasets

Nineteen variables identified as risk factors for GC were incorporated into the prediction model, namely, age; sex; body mass index (BMI); health insurance premium; smoking status; alcohol consumption; family history of GC, colorectal cancer, or liver cancer; and personal history of hypertension, diabetes mellitus, myocardial infarction/angina pectoris, stroke, dyslipidemia, colorectal cancer, liver cancer, *H. pylori* infection, gastric adenoma, and AG or IM. These variables were pre-specified *a priori* based on guideline statements and epidemiologic evidence for gastric cancer risk in Asian screening contexts, supplemented by lifestyle and clinical-context variables available with high completeness/validity in NHID/NCSP. Specifically, we grouped predictors as: (i) guideline-anchored risk factors—*H. pylori* eradication history, AG/IM, age, sex, smoking, and family history of GC; (ii) lifestyle—BMI and alcohol; and (iii) contextual clinical variables—socioeconomic status (insurance premium), cardiometabolic comorbidities (hypertension, diabetes, dyslipidemia, myocardial infarction/angina, stroke), and prior cancers (colorectal, liver). Comorbidities and prior cancers were incorporated as non-causal context markers of baseline health and health-care pathways rather than as prespecified causal determinants (**Supplementary Table 1**).

Age groups were divided into 5-year intervals, and BMI was categorized as normal (<23 kg/m²), overweight (23.0–24.9), obese (25.0–29.9), and severely obese (≥30.0), consistent with Korean/Asia-Pacific criteria (19, 20). Economic status was classified into five groups based on health insurance premiums (medical aid beneficiaries and quartiles 1–4). Smoking status was categorized as nonsmoker, former smoker, or current smoker, and alcohol consumption was classified as nondrinker, drinking three or fewer times per week, or drinking four or more times per week. Alcohol exposure was available only as frequency categories in the 2018 NHID/NCSP screening dataset (nondrinker; ≤3 times/week; ≥4 times/week); per-occasion quantity (standard drinks) and grams of ethanol were not recorded in the analytic extract and thus could not be estimated. Family and medical histories were dichotomized as either present or absent. A history of *H. pylori* infection was defined based on records of the prescribed eradication regimens. Gastric adenoma was confirmed by gastroscopy and biopsy results from GC screening.

The dataset was divided into a training set and a test set in an 8:2 ratio. The Cox proportional hazard model (CPHM) and three machine learning-based prediction models, namely, logistic regression (LR), decision tree (DT), and extreme Gradient Boosting (XGBoost), were developed. Hyperparameter tuning for the LR and DT models was performed using five-fold cross-validation on the training set, whereas the boosting iteration for XGBoost was set to 500. To mitigate overfitting in the XGBoost model, we applied both L1 (reg_alpha = 1) and L2 (reg_lambda = 1) regularization, in addition to a low learning rate (η = 0.01), restricted tree depth (max_depth = 5), and subsampling (subsample = 0.8). Early stopping was implemented by monitoring the evaluation loss on a validation set (early_stopping_rounds = 50). Although more extensive hyperparameter optimization such as nested cross-validation could further improve model performance, it could not be implemented due to computational constraints. Accordingly, the maximum number of boosting rounds was fixed at 500, and the early-stopping procedure adaptively determined the effective number of iterations based on validation performance. Model performance was assessed using the accuracy, sensitivity, specificity, and area under the receiver operating characteristic curve (AUROC).

### 2.3 Analyses

We conducted three sensitivity analyses:

(1) multiple imputations of missing covariates using the Multivariate Imputation by Chained Equations (MICE) package (m=5); (2) feature selection using least absolute shrinkage and selection operator (LASSO) regression to remove potentially redundant factors and reduce the risk of data overfitting (21); and (3) missing imputation with feature selection.

External validation was conducted using the Cancer Screenee Cohort Study of the National Cancer Center, a dataset comprising of 31,285 individuals. This cohort was used to evaluate the generalizability and predictive performance of the models using an independent dataset.

To improve the interpretability of the top-performing model (with the highest AUROC value in the external validation set), the Shapley Additive Explanations (SHAP) algorithm was incorporated. This game theory-based method elucidates the contribution of each feature to model predictions, overcoming challenges related to the opacity of machine learning models (22). To support reproducibility and practical implementation, the full R code used to develop and validate the XGBoost model has been included in **Supplementary Appendix 1**.

All analyses were conducted using R software (version 3.6.4), adhering to the STROBE reporting guidelines.

### 2.4 Ethical considerations

This study was approved by the Institutional Review Board of the National Cancer Center (Approval number: Ncc2021-0141). The requirement for informed consent was waived because the data analyses were performed retrospectively using anonymized data.

## 3 Results

**Figure 1:**
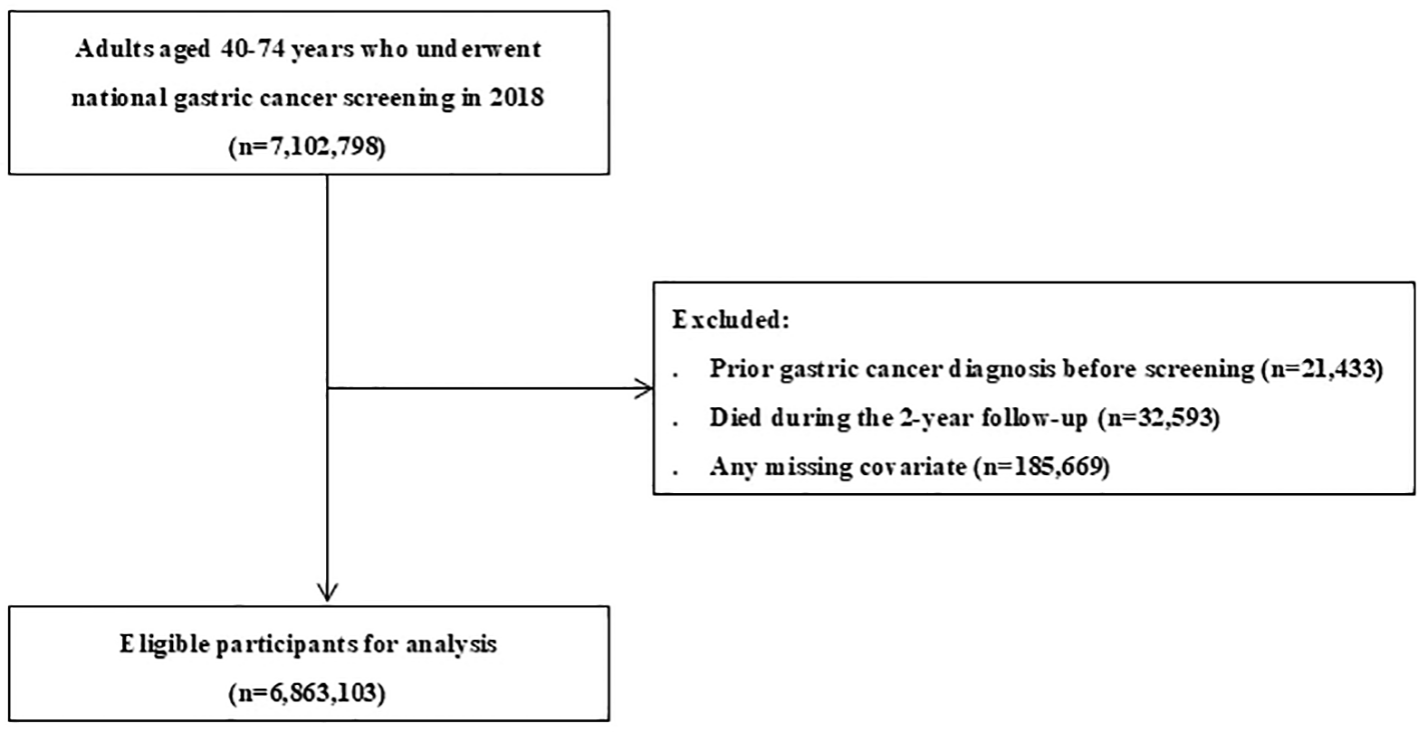
Flow chart of the study participants.

A total of 6,863,103 individuals were included in the study, comprising 2,624 with incident GC during the 2-year follow-up and 6,860,479 without incident GC (**Figure 1**). For model development, the cohort was randomly divided into training (80%) and test (20%) sets, yielding 5,490,482 individuals in the training set (2,099 GC cases and 5,488,383 non-GC cases) and 1,372,621 individuals in the test set (525 GC cases and 1,372,096 non-GC cases). The characteristics of the study participants are summarized in **Table 1**. Compared to those without incident GC, participants who developed GC were older and more often male (73.3% vs. 46.5%, *p* < 0.001). The BMI distribution differed, with obesity (≥30 kg/m²) being less common among patients with GC (3.6% vs. 5.3%, *p* < 0.001). The distribution of the health insurance premium categories did not differ significantly (*p* = 0.178). Smoking status varied markedly (current smoking 31.6% vs. 17.9%; ex-smoking 26.9% vs. 18.6%; *p* < 0.001), and drinking frequency also differed (≥4 times/week 6.6% vs. 8.7%, *p* < 0.001). The family history of GC was more frequent among patients with GC (13.9% vs. 9.7%, *p* < 0.001). Hypertension and hyperlipidemia were less prevalent among patients with GC (both *p* < 0.001), and the prevalence of diabetes was similar (*p* = 0.076). Preexisting myocardial infarction/angina, stroke, colorectal cancer, and liver cancer did not differ significantly between the groups (all *p*>0.3). In contrast, *H. pylori* infection, AG/IM, and gastric adenoma were more frequent among patients with GC (all *p* < 0.001).

**Table 1:** Baseline characteristics of the study population.

Characteristics	Total (n=6,863,103)	No gastric cancer (n=6,860,479)	Gastric cancer (n=2,624)	*P*-value
Age				<0.001
40–44	1,156,886 (16.9)	1,156,739 (16.9)	147 (5.6)	
45–49	923,474 (13.5)	923,282 (13.5)	192 (7.3)	
50–54	1,284,123 (18.7)	1,283,702 (18.7)	421 (16.0)	
55–59	997,762 (14.5)	997,270 (14.5)	492 (18.8)	
60–64	1,203,969 (17.5)	1,203,348 (17.5)	621 (23.7)	
65–69	650,132 (9.5)	649,770 (9.5)	362 (13.8)	
70–74	646,757 (9.4)	646,368 (9.4)	389 (14.8)	
Sex				<0.001
Male	3,193,616 (46.6)	3,191,692 (46.5)	1,924 (73.3)	
Female	3,669,487 (53.4)	3,668,787 (53.5)	700 (26.7)	
BMI (kg/m^2^)				<0.001
<23	2,433,275 (35.5)	2,432,286 (35.5)	989 (37.7)	
23–24.9	1,739,760 (25.4)	1,739,087 (25.3)	673 (25.6)	
25–29.9	2,329,619 (33.9)	2,328,751 (33.9)	868 (33.1)	
≥30	360,449 (5.2)	360,355 (5.3)	94 (3.6)	
Health insurance premium				0.178
Medical Aid beneficiaries	248,804 (3.7)	248,719 (3.6)	85 (3.2)	
~25 percentile	1,347,019 (19.6)	1,346,512 (19.6)	507 (19.3)	
~50 percentile	1,322,785 (19.2)	1,322,251 (19.3)	534 (20.4)	
~75 percentile	1,646,985 (24.0)	1,646,324 (24.0)	661 (25.2)	
~100 percentile	2,297,510 (33.5)	2,296,673 (33.5)	837 (31.9)	
Smoking				<0.001
Non-Smoker	4,357,937 (63.5)	4,356,847 (63.5)	1,090 (41.5)	
Ex-Smoker	1,273,595 (18.6)	1,272,889 (18.6)	706 (26.9)	
Smoker	1,231,571 (17.9)	1,230,743 (17.9)	828 (31.6)	
Drinking				<0.001
No drinking	4,703,278 (68.5)	4,701,446 (68.5)	1,832 (69.8)	
≤ 3 times/week	1,564,668 (22.8)	1,564,049 (22.8)	619 (23.6)	
≥ 4 times/week	595,157 (8.7)	594,984 (8.7)	173 (6.6)	
Family history of gastric cancer				<0.001
No	6,199,918 (90.3)	6,197,659 (90.3)	2,259 (86.1)	
Yes	663,185 (9.7)	662,820 (9.7)	365 (13.9)	
Family history of colorectal cancer				0.317
No	6,595,230 (96.1)	6,592,698 (96.1)	2,532 (96.5)	
Yes	267,873 (3.9)	267,781 (3.9)	92 (3.5)	
Family history of liver cancer				0.662
No	6,553,544 (95.5)	6,551,043 (95.5)	2,501 (95.3)	
Yes	309,559 (4.5)	309,436 (4.5)	123 (4.7)	
Hypertension				<0.001
No	6,317,180 (92.1)	6,314,686 (92.0)	2,494 (95.0)	
Yes	545,923 (7.9)	545,793 (8.0)	130 (5.0)	
Diabetes				0.076
No	6,195,035 (90.3)	6,192,639 (90.3)	2,396 (91.3)	
Yes	668,068 (9.7)	667,840 (9.7)	228 (8.7)	
Hyperlipidemia				<0.001
No	6,231,934 (90.8)	6,229,485 (90.8)	2,449 (93.3)	
Yes	631,169 (9.2)	630,994 (9.2)	175 (6.7)	
Myocardial infarction or angina pectoris				0.351
No	6,770,177 (98.6)	6,767,583 (98.6)	2,594 (98.9)	
Yes	92,926 (1.4)	92,896 (1.4)	30 (1.1)	
Stroke				0.448
No	6,794,728 (99.0)	6,792,134 (99.0)	2,594 (98.9)	
Yes	68,375 (1.0)	68,345 (1.0)	30 (1.1)	
Colorectal cancer				0.353
No	6,839,054 (99.6)	6,836,442 (99.6)	2,612 (99.5)	
Yes	24,049 (0.4)	24,037 (0.4)	12 (0.5)	
Liver cancer				0.828
No	6,853,711 (99.9)	6,851,091 (99.9)	2,620 (99.8)	
Yes	9,392 (0.1)	9,388 (0.1)	4 (0.2)	
*Helicobacter pylori* infection				<0.001
No	6,834,934 (99.6)	6,832,378 (99.6)	2,556 (97.4)	
Yes	28,169 (0.4)	28,101 (0.4)	68 (2.6)	
Atrophic gastritis or intestinal metaplasia				<0.001
No	6,536,623 (95.2)	6,535,105 (95.3)	1,518 (57.9)	
Yes	326,480 (4.8)	325,374 (4.7)	1,106 (42.1)	
Gastric adenoma				<0.001
No	6,845,012 (99.7)	6,842,962 (99.7)	2,050 (78.1)	
Yes	18,091 (0.3)	17,517 (0.3)	574 (21.9)	

Values are presented as number (%).

BMI, body mass index.

In the external validation cohort (Cancer Screenee Cohort, n = 31,285), demographic and endoscopic patterns among gastric cancer cases were consistent with those observed in the development dataset. Gastric cancer cases were predominantly in their 50s–60s and more frequently male. The prevalences of atrophic gastritis/intestinal metaplasia and gastric adenoma were also markedly higher among cases than non-cases. Detailed characteristics of the cohort are presented in **Supplementary Table 2**.

**Figure 2:**
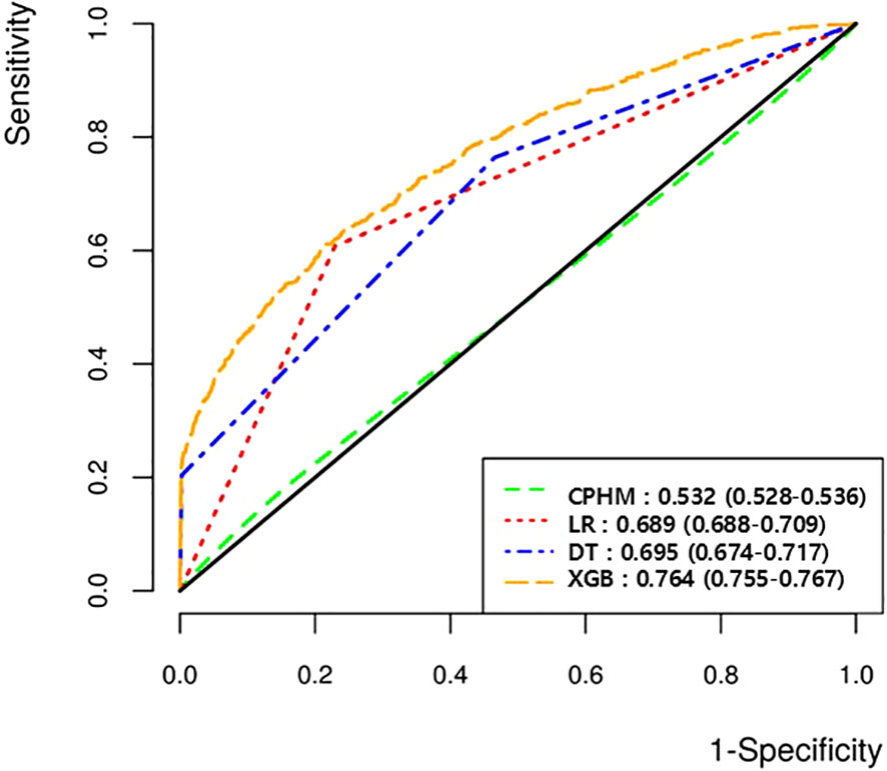
AUROCs of the prediction model. AUROC, area under the receiver operating characteristic curve; CPHM, cox proportional hazards model; LR, logistic regression; DT, decision tree; XGBoost, eXtreme Gradient Boosting.

The performances of the models are summarized in **Table 2**. In the internal validation set, XGBoost had the highest AUROC (0.764; 95% confidence interval [CI], 0.755–0.767) (**Figure 2**) and specificity (0.746; 95% CI, 0.740–0.750). DT achieved the highest sensitivity (0.764, 95% CI, 0.730–0.800), whereas LR had the highest accuracy (0.770, 95% CI, 0.769–0.771). CPHM performed the worst across all metrics (AUROC 0.532, 95% CI, 0.528–0.536). In the external validation set, XGBoost had the highest AUROC (0.708; 95% CI, 0.682–0.884). In the sensitivity analyses, the results were consistent across approaches, with XGBoost retaining the highest AUROC (**Supplementary Figure 1**).

**Table 2:** Performance of CPHM and different machine learning models without missing imputation.

	Accuracy	Sensitivity	Specificity	AUROC
Internal validation set				
CPHM	0.534 (0.531–0.537)	0.531 (0.529–0.533)	0.511 (0.510–0.512)	0.532 (0.528–0.536)
LR	0.770 (0.769–0.771)	0.607 (0.560–0.650)	0.700 (0.693–0.704)	0.689 (0.668–0.709)
DT	0.695 (0.674–0.717)	0.764 (0.730–0.800)	0.534 (0.530–0.540)	0.695 (0.674–0.717)
XGBoost	0.746 (0.744–0.750)	0.607 (0.560–0.650)	0.746 (0.740–0.750)	0.764 (0.755–0.767)
External validation set				
CPHM	0.631 (0.628–0.634)	0.556 (0.555–0.557)	0.706 (0.703–0.709)	0.611 (0.599–0.613)
LR	0.425 (0.420–0.431)	0.700 (0.669–0.708)	0.425 (0.420–0.430)	0.512 (0.423–0.602)
DT	0.655 (0.578–0.736)	0.800 (0.610–0.920)	0.483 (0.480–0.490)	0.655 (0.578–0.736)
XGBoost	0.597 (0.592–0.603)	0.666 (0.470–0.830)	0.597 (0.590–0.600)	0.708 (0.682–0.884)

Values in parentheses represent 95% confidence intervals. AUROC, area under the receiver operating characteristic curve; CPHM, Cox proportional hazards model; LR, logistic regression; DT, decision tree; XGBoost, extreme Gradient Boosting.

**Figure 3:**
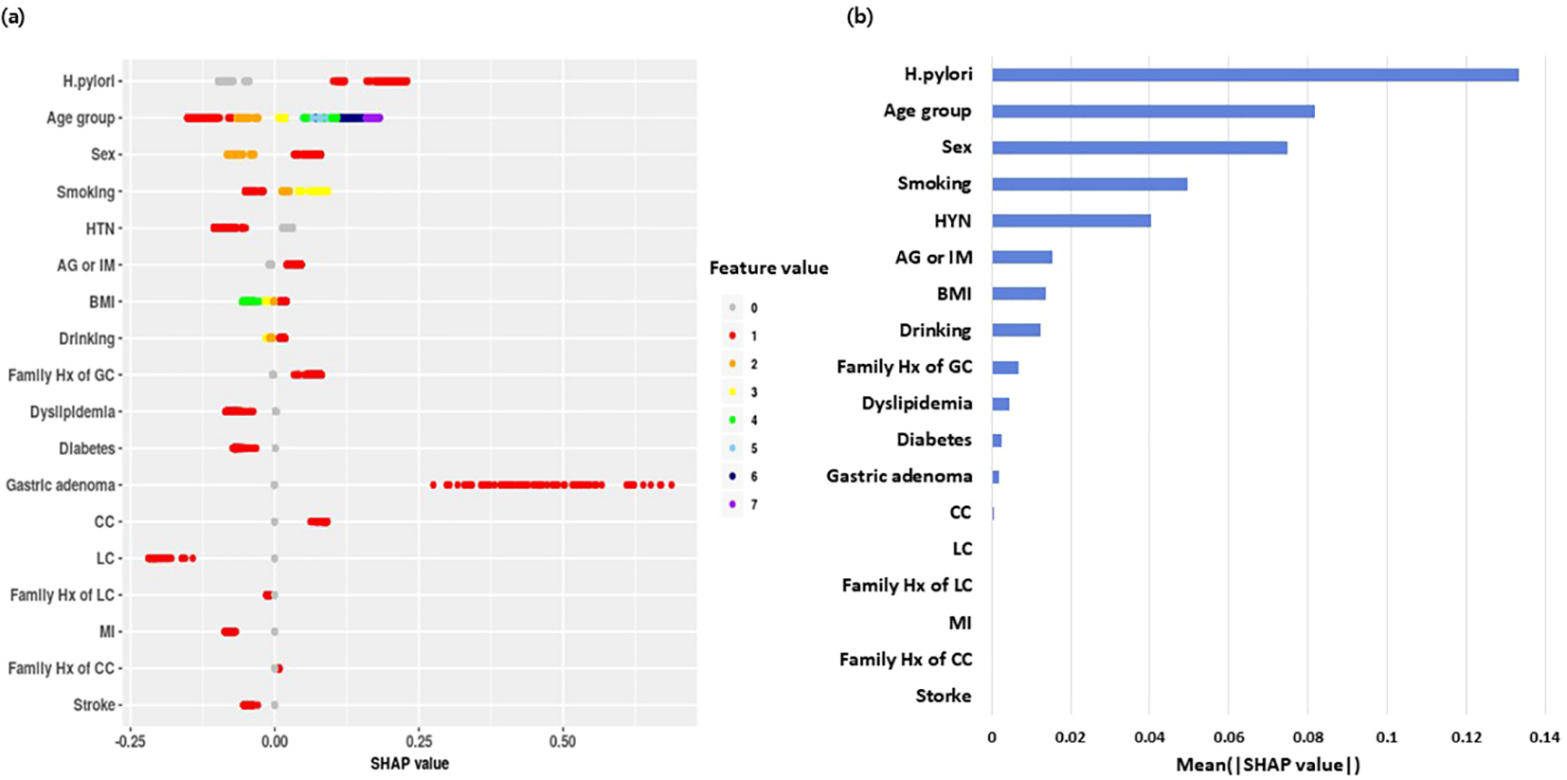
SHAP summary plots for predictors of incident gastric cancer. **(a)** Beeswarm plot of SHAP values for 19 predictors in the external validation set. Each dot represents one participant, with SHAP values on the x-axis and feature values encoded by color. Positive SHAP values increase the predicted risk, and negative values decrease the predicted risk. **(b)** Global importance based on mean absolute SHAP. Longer bars indicate larger average contributions.

**Figure 3** presents the SHAP explanations for the final XGBoost model, which is the model showing the best discrimination in the external validation set. Panel (A) shows a beeswarm plot of individual SHAP values (x-axis) with feature values encoded by color; positive values increase and negative values decrease the predicted GC risk. Panel (B) ranks the predictors by global importance using the mean absolute SHAP value. The largest contributions were from *H. pylori* infection, age, sex, smoking status, and AG/IM, followed by BMI and a family history of GC. Specifically, *H. pylori* infection, older age, male sex, current smoking, and the AG/IM ratio showed positive SHAP contributions, indicating a higher predicted GC risk. In contrast, the presence of hypertension showed negative SHAP contributions, consistent with a lower predicted risk.

## 4 Discussion

In a large real-world Asian screening cohort, we showed that a 2-year GC risk model explicitly integrating AG/IM with demographic and lifestyle factors may inform risk-adapted endoscopic surveillance. We compared CPHM with machine-learning approaches (i.e., XGBoost, DT, and LR). XGBoost achieved the highest AUROC in both the internal and external validations, whereas CPHM showed the lowest performance. Beyond discrimination, SHAP quantified each predictor’s contribution and rendered the model clinically transparent. A history of *H. pylori* infection, age, sex, smoking, and AG/IM were the dominant positive contributors. At the individual level, SHAP generated explanation profiles showing how specific features increased or decreased an individual’s predicted risk, which could support targeted counseling and triage in future implementations. By drawing on mucosal pathology aligned with the Correa cascade, this study advances implementable, biology-informed stratification for high-burden Asian settings.

GC has a high disease burden, ranking 5th worldwide for both cancer incidence and mortality (1). Recent global projections highlight the substantial and growing disease burden of GC, with a projected lifetime burden for the 2008–2017 birth cohorts of approximately 15.6 million cases (~76% *H. pylori*-attributable). With a burden expected to double in the Americas and rise six-fold in sub-Saharan Africa, there would be potential value in pairing endoscopic screening with risk-stratified, personalized strategies anchored to *H. pylori* and high-risk endoscopic findings (AG, IM) (23, 24). High-performance prediction models can help identify individuals at elevated risk and support tailored surveillance and intervention plans. In our study, the XGBoost model maintained strong discrimination in external validation, underscoring its potential clinical utility.

According to the Correa hypothesis, intestinal-type GC occurs preferentially in the presence of AG and IM (7). Therefore, appropriate treatment and longitudinal management of these precursor lesions are important public health priorities in Korea, where the incidence of GC is high. In addition, the current Korean endoscopy guidelines recommend biennial screening endoscopy for adults aged ≥40 years; however, there is an ongoing debate as to whether patients with IM and/or AG should undergo surveillance at intervals shorter than 2 years. The 2020 AGA Clinical Practice Guidelines (Gastroenterology) estimated the annual risk of progression from IM to GC to be 0.124%, whereas a Korean study reported a higher annual progression risk of 0.187% in the presence of IM (25). Furthermore, a study that followed up patients with IM found progression to adenoma or GC after a median of 11–32 months, with incomplete-type IM tending to progress more rapidly (26). Collectively, these findings suggest that surveillance at intervals shorter than 2 years may benefit patients with incomplete-type IM; however, confirmatory evidence from Korean cohorts is currently lacking.

Based on the first national screening dataset in which AG/IM endoscopic codes were available, our study incorporated AG/IM findings into a 2-year GC risk-prediction model and identified these features as risk-increasing factors. These findings may inform the consideration of a shift from uniform biennial endoscopy to biology-informed, risk-stratified scheduling in high-burden Asian screening programs. The results provide external validation and SHAP-based interpretability for programmatic adoption and patient-facing communication. Although our analysis could not directly evaluate the optimal surveillance interval, future studies are warranted to determine whether endoscopic screening at intervals of less than 2 years in patients with AG/IM reduces GC mortality.

The proposed models could be used to (1) flag high-risk individuals in routine screening programs and (2) inform personalized risk communication and management. SHAP-based explanations may guide tailored interventions by highlighting modifiable contributors (e.g., *H. pylori* eradication and smoking cessation) and contextualizing AG/IM-driven risk in shared decision-making regarding surveillance timing.

A key strength of this study is the large real-world dataset derived from the National Health Insurance Service program, which includes robust demographics, diagnostic codes, medication use, and endoscopic results implicating high-risk findings such as AG, IM, and gastric adenoma. Notably, AG or IM coding has been available since 2018, enabling one of the first short-term GC prediction analyses to explicitly incorporate these high-risk endoscopic features. Another strength is our use of SHAP to mitigate the “black-box” limitation of machine-learning models by clarifying the direction and magnitude of each variable’s effect.

The primary limitation of this study is the relatively short follow-up period, which restricts inferences about short-term (2-year) GC risk. Longer follow-up and prospective evaluations are needed to assess calibration drift, clinical impact, and whether intensified surveillance for AG/IM reduces GC incidence and mortality. Because survival-based machine-learning models could not be implemented within the NHIS secure analysis environment, we were unable to perform a conceptually consistent comparison between Cox and survival-ML approaches. Therefore, we compared the Cox proportional hazards model with available binary classification algorithms, using Cox as the traditional and most widely used benchmark in clinical and epidemiologic prediction research. These analytical constraints also influenced our handling of censoring, and we excluded individuals who died within two years after screening to minimize outcome misclassification. Although this approach reduced misclassification of early deaths, it may have introduced selection or survivorship bias and informative censoring, potentially attenuating estimated risks.

Another limitation is that we restricted the cohort to individuals aged 40–74 years, which limits generalizability to adults ≥75 years. In addition, our dataset captured alcohol frequency but not per-occasion quantity or ethanol grams, precluding dose–response analyses and potentially attenuating alcohol-related effects. Moreover, our evaluation focused primarily on discrimination metrics such as AUROC, accuracy, sensitivity, and specificity; although this approach is consistent with prior gastric cancer prediction studies (27), the lack of calibration assessment and clinical-utility evaluation using decision curve analysis (DCA) remains a limitation.

Finally, the closed and fixed-version computational environment substantially limited the scope of hyperparameter tuning. This restriction may have constrained achievable discrimination, calibration performance, and overall model stability; broader hyperparameter optimization may yield improved performance in settings with more flexible computational infrastructure.

## 5 Conclusions

Using a large, national screening cohort including high-risk endoscopic findings, we developed a machine-learning model, XGBoost, with higher discrimination than that of conventional approaches for predicting 2-year GC risk. Beyond identifying high-risk individuals, the SHAP-based interpretability facilitates personalized counseling and targeted interventions. Although our results support the rationale for risk-adapted surveillance, potentially with shorter intervals for patients with AG/IM, prospective studies are warranted to determine the optimal surveillance frequency and its effect on GC outcomes.

## Data Availability

Data cannot be shared publicly because the data are from the Korean National Health Insurance Service (NHIS) health screening cohort and are subject to access restrictions. Data are available from the NHIS Institutional Data Access/Ethics Committee for qualified researchers who meet the criteria for access to confidential data. Contact: URL: https://nhiss.nhis.or.kr/; Address: 32 Gungang-ro, Wonju-si, Gangwon-do 26464, Republic of Korea. Requests to access the datasets should be directed to https://nhiss.nhis.or.kr.
