# Supplementary material for: SHAP-explained machine-learning model for high-risk gastric cancer identification
Source: Front Oncol. 2026 Mar 16;16:1732072. doi: 10.3389/fonc.2026.1732072 (PMC13033554; doi:10.3389/fonc.2026.1732072)
Supplement: Supplementary file 1 [file DataSheet1.docx]

Supplementary Appendix 1. R Code for Reproducible Implementation of the Best-Performing Extreme Gradient Boosting Model

This appendix provides the complete R code used for data preprocessing, development of the best-performing prediction model based on Extreme Gradient Boosting (XGBoost), class imbalance correction using Random Over-Sampling Examples (ROSE), and internal validation through the area under the receiver operating characteristic curve (AUROC).

**R Code :**

################################################################################

# Dataset Preparation for 2-Year Gastric Cancer Incidence Analysis

################################################################################

# Variable selection and factor conversion

Gastric_cancer <- Gastric_cancer %>% dplyr::select(

SEX, AGE_type, BMI_type, HP, AD, ag_im,

cancer_2yr_outcome, cancer_2yr_outcome_time,

coc, lc,

x1, x2, x3, x4, x5,

fm1, fm2, fm3,

sm_value, alcohol

) %>%

mutate_at(vars(

SEX, AGE_type, BMI_type, HP, AD, ag_im,

coc, lc,

x1, x2, x3, x4, x5,

fm1, fm2, fm3,

sm_value, alcohol

), as.factor)

# Binary outcome variables converted to numeric (0/1 coding)

Gastric_cancer$cancer_2yr_outcome <- as.numeric(Gastric_cancer$cancer_2yr_outcome)

Gastric_cancer$cancer_2yr_outcome_time <- as.numeric(Gastric_cancer$cancer_2yr_outcome_time)

################################################################################

# Variable Definitions

################################################################################

# SEX : Male / Female

# AGE_type : Age (40–44, 45–49, 50–54, 55–59, 60–64, 65–69, 70–74)

# BMI_type : BMI (kg/m^2): <23, 23–24.9, 25–29.9, ≥30

# sm_value : Smoking (Never / Former / Current)

# alcohol : Drinking frequency (No / ≤3/week / ≥4/week)

# fm1~fm3 : Family history (GC, CRC, Liver cancer) – No / Yes

# x1~x5 : Comorbidities (HTN, DM, Dyslipidemia, MI/Angina, Stroke) – No / Yes

# HP : Helicobacter pylori infection – No / Yes

# AD : Atrophic gastritis or intestinal metaplasia – No / Yes

# ag_im : Premalignant gastric lesions – No / Yes

# coc : Colorectal cancer comorbid condition – No / Yes

# lc : Liver cancer comorbid condition – No / Yes

# cancer_2yr_outcome : GC incidence within 2 years (0 / 1)

# cancer_2yr_outcome_time : Follow-up time (days)

################################################################################

# Train–Test Split (80% / 20%)

################################################################################

set.seed(123)

ind <- sample(2, nrow(Gastric_cancer), replace = TRUE, prob = c(0.8, 0.2))

train_set <- Gastric_cancer[ind == 1, ]

test_set <- Gastric_cancer[ind == 2, ]

################################################################################

# Random Over-Sampling (ROSE) to Address Class Imbalance

################################################################################

library(ROSE)

train_set_sampling <- ROSE(cancer_2yr_outcome ~ ., data = train_set, seed = 123)$data

################################################################################

# Machine Learning Model: XGBoost with Early Stopping

################################################################################

library(xgboost)

library(pROC)

# Convert target variable to numeric 0/1

train_label <- as.numeric(train_set_sampling$cancer_2yr_outcome) - 1

test_label <- as.numeric(test_set$cancer_2yr_outcome) - 1

# One-hot encoding for categorical predictors

x_train <- model.matrix(cancer_2yr_outcome ~ . - 1, data = train_set_sampling)

x_test <- model.matrix(cancer_2yr_outcome ~ . - 1, data = test_set)

# Convert to DMatrix format

dtrain <- xgb.DMatrix(data = as.matrix(x_train), label = train_label)

dtest <- xgb.DMatrix(data = as.matrix(x_test), label = test_label)

# Hyperparameter setup with regularization and subsampling

param <- list(

objective = "binary:logistic",

eval_metric = "auc",

eta = 0.01,

max_depth = 5,

subsample = 0.8,

reg_alpha = 1,

reg_lambda = 1,

min_child_weight = 1

)

# Model Training

set.seed(123)

xgb_fit <- xgb.train(

params = param,

data = dtrain,

nrounds = 500,

watchlist = list(valid = dtest),

early_stopping_rounds = 50,

print_every_n = 50,

verbose = 1

)

################################################################################

# Performance Evaluation (AUROC)

################################################################################

pred_prob <- predict(xgb_fit, dtest)

ROC_xgb <- roc(test_label, pred_prob)

AUC_xgb <- auc(ROC_xgb)

AUC_xgb # AUROC value

plot(ROC_xgb, main = "ROC Curve for XGBoost (2-Year Gastric Cancer Prediction)")
