# Supplementary figures and images for: SHAP-explained machine-learning model for high-risk gastric cancer identification
Source: Front Oncol. 2026 Mar 16;16:1732072. doi: 10.3389/fonc.2026.1732072 (PMC13033554; doi:10.3389/fonc.2026.1732072)

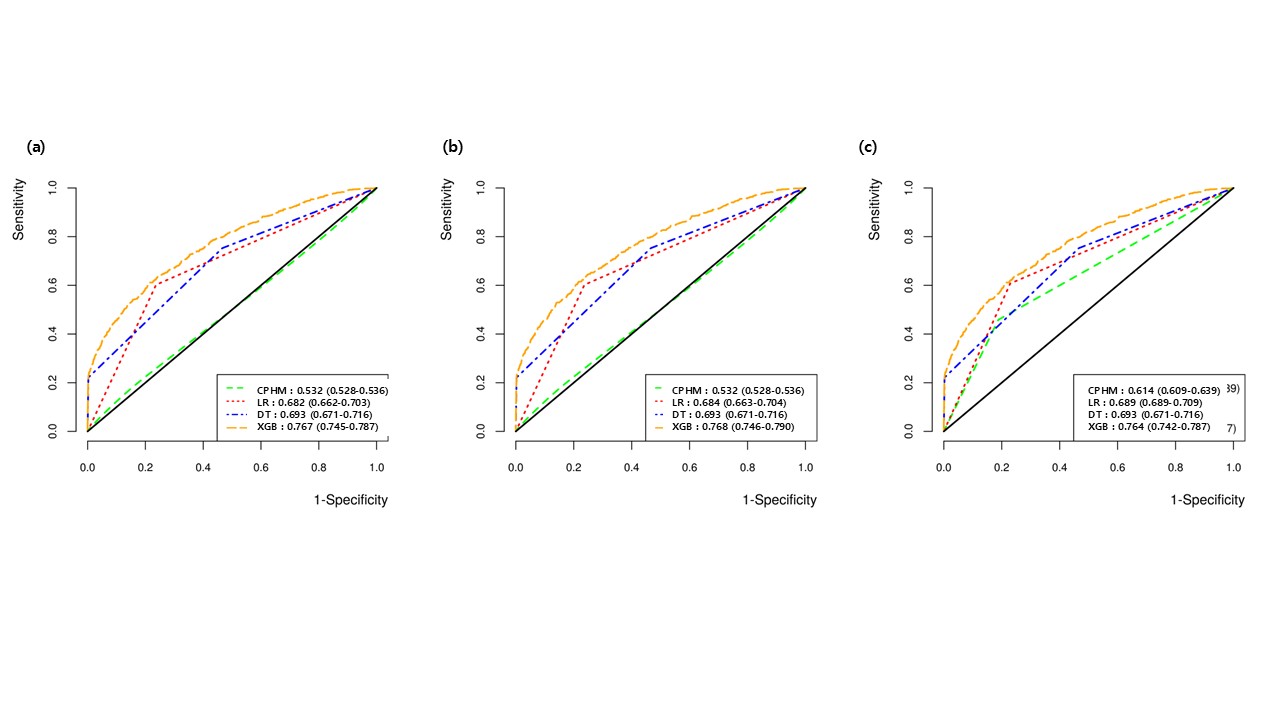

Supplement: Supplementary file 2 [file Image1.jpeg]
