# Supplementary material for: SHAP-explained machine-learning model for high-risk gastric cancer identification
Source: Front Oncol. 2026 Mar 16;16:1732072. doi: 10.3389/fonc.2026.1732072 (PMC13033554; doi:10.3389/fonc.2026.1732072)
Supplement: Supplementary file 3 [file Table1.docx]

**Supplementary Table S1 - Evidence for Predictor Selection**

This table lists predictors, their operationalization, and evidence with numbered references corresponding to the list below.

| **Predictor** | **Operationalization in this study (per Methods)** | **Evidence rationale (abridged)** | **Key refs [#]** |
| --- | --- | --- | --- |
| Age (5-year groups) | Age at index NCSP endoscopy in 2018; grouped 40-44 / 45-49 / 50-54 / 55-59 / 60-64 / 65-69 / 70-74. | Gastric cancer risk rises with age; standard covariate in epidemiology and guidelines. | [1] |
| Sex | Administrative sex (male/female). | Male sex associated with higher incidence and mortality in global data. | [1] |
| Body mass index (BMI) | Measured at screening; categorized <23, 23-24.9, 25-29.9, >=30 kg/m2 (Asia-Pacific cutpoints). | Asian BMI thresholds recommended; adiposity relates to cardia risk and metabolic milieu. | [8,9] |
| Health insurance premium (SES proxy) | Medical Aid plus quartiles 1-4 from NHIS eligibility records. | Validated proxy for socioeconomic status in Korean NHID studies. | [10,11] |
| Smoking status | Self-reported: never, former, current. | Cohort meta-analyses show dose-response increase in gastric cancer risk among smokers. | [6] |
| Alcohol consumption (frequency) | Self-reported frequency: none; <=3 times/week; >=4 times/week. | Heavy or frequent intake associated with higher gastric cancer risk in meta-analyses. | [7] |
| Family history of gastric cancer | First-degree family history (yes/no). | Pooled analyses show approximately 2-fold increased risk with FDR history. | [5] |
| Family history of colorectal cancer | First-degree family history (yes/no). | Direct association with gastric cancer is limited; retained as proxy for shared GI susceptibility. | [12] |
| Family history of liver cancer | First-degree family history (yes/no). | Direct association with gastric cancer is limited; contextual familial exposure marker. | [12] |
| Hypertension (history) | Binary history flag (claims or questionnaire). | Included as comorbidity/context variable; mixed associations with gastric cancer incidence. | [13,16] |
| Diabetes mellitus (history) | Binary history flag (claims or questionnaire). | Some studies suggest modestly higher risk; included as part of metabolic profile. | [13] |
| Dyslipidemia (history) | Binary history flag (claims or questionnaire). | Heterogeneous evidence by lipid fraction; represents metabolic milieu. | [13] |
| Myocardial infarction or angina (history) | Binary history flag (claims). | Comorbidity burden rather than causal driver; affects care pathways and ascertainment. | [16] |
| Stroke (history) | Binary history flag (claims). | Comorbidity/context variable; may influence utilization and outcomes. | [16] |
| Personal history of colorectal cancer | Binary prior cancer flag (claims). | Represents oncologic history and shared risk or surveillance intensity. | [12,16] |
| Personal history of liver cancer | Binary prior cancer flag (claims). | Context variable; not assumed strong causal driver of gastric cancer risk. | [12,16] |
| Helicobacter pylori infection (history) | Claims-based ascertainment via eradication regimen prescriptions. | Established causal risk factor for non-cardia gastric cancer; claims-based definitions validated. | [4,19] |
| Atrophic gastritis or intestinal metaplasia (AG/IM) | Endoscopic diagnosis codes (available since 2018). | Precancerous mucosal conditions in the Correa cascade; guideline-endorsed risk markers. | [2,3] |
| Gastric adenoma | Biopsy-confirmed adenoma at screening endoscopy. | Part of dysplasia spectrum; post-resection cohorts show elevated metachronous risk. | [14,15] |

**References (numbered, Vancouver style)**

[1] Bray F, Laversanne M, Sung H, Ferlay J, Siegel RL, Soerjomataram I, et al. Global cancer statistics 2022. CA Cancer J Clin. 2024;74:229-263. doi:10.3322/caac.21834.

[2] Gawron AJ, Shah SC, Altayar O, Davitkov P, Morgan D, Turner K, et al. AGA technical review on gastric intestinal metaplasia. Gastroenterology. 2020;158:732-744. doi:10.1053/j.gastro.2019.12.003.

[3] Dinis-Ribeiro M, Libanio D, Uchima H, Spaander MC, Bornschein J, Matysiak-Budnik T, et al. MAPS III guideline update 2025. Endoscopy. 2025;57:504-554. doi:10.1055/a-2529-5025.

[4] Gu J, He F, Clifford GM, Li M, Fan Z, Li X, et al. H. pylori and gastric cancer risk: meta-analysis. Expert Rev Mol Diagn. 2023;23:1251-1261. doi:10.1080/14737159.2023.2277377.

[5] Vitelli-Storelli F, Rubin-Garcia M, Pelucchi C, Benavente Y, Bonzi R, Rota M, et al. Family history and gastric cancer risk: StoP pooled analysis. Cancers (Basel). 2021;13:3844. doi:10.3390/cancers13153844.

[6] Ladeiras-Lopes R, Pereira AK, Nogueira A, Pinheiro-Torres T, Pinto I, Santos-Pereira R, Lunet N. Smoking and gastric cancer: cohort meta-analysis. Cancer Causes Control. 2008;19:689-701. doi:10.1007/s10552-008-9132-y.

[7] Tramacere I, Negri E, Pelucchi C, Bagnardi V, Rota M, Scotti L, et al. Alcohol and gastric cancer risk: meta-analysis. Ann Oncol. 2012;23:28-36. doi:10.1093/annonc/mdr135.

[8] WHO Expert Consultation. Appropriate BMI for Asian populations. Lancet. 2004;363:157-163. doi:10.1016/S0140-6736(03)15268-3.

[9] WHO Western Pacific Region. The Asia-Pacific perspective: Redefining obesity and its treatment. 2000.

[10] Bahk J, Kim YY, Kang H-Y, Lee J, Kim I, Lee J, et al. Using the NHID for monitoring mortality and life expectancy. J Korean Med Sci. 2017;32:1764-1770. doi:10.3346/jkms.2017.32.11.1764.

[11] Seong SC, Kim YY, Khang YH, Park JH, Kang H-J, Lee H, et al. Data resource profile: NHID of NHIS in Korea. Int J Epidemiol. 2017;46:799-800. doi:10.1093/ije/dyw253.

[12] Ko KP, Kim Y, Park B. Risk factors of gastric cancer and lifestyle modification: updated review. J Gastric Cancer. 2024;24:e10. doi:10.5230/jgc.2024.24.e10.

[13] Mariani M, Pastorino R, Pavesi E, Copetti M, Boccia S. Metabolic syndrome and gastric cancer risk: systematic review and meta-analysis. Cancer Epidemiol. 2021;72:101938. doi:10.1016/j.canep.2021.101938.

[14] Yoon SB, Park JM, Lim CH, Lee IS, Choi MG, Chun HJ, et al. Incidence of gastric cancer after endoscopic resection of gastric adenoma and early GC. Gastrointest Endosc. 2016;83:1176-1183. doi:10.1016/j.gie.2015.10.034.

[15] Park JM, Kim EH, Kim JH, Lee H, Jung DH, et al. Gastric cancer after removal of gastric adenomas: nationwide cohort. Am J Gastroenterol. 2023;118:1542-1553. doi:10.14309/ajg.0000000000002418.

[16] Charlson ME, Pompei P, Ales KL, MacKenzie CR. Classifying prognostic comorbidity: development and validation. J Chronic Dis. 1987;40:373-383. doi:10.1016/0021-9681(87)90171-8.

[17] Nagtegaal ID, Odze RD, Klimstra D, Paradis V, Rugge M, Schirmacher P, et al. 2019 WHO classification of digestive system tumours. Histopathology. 2020;76:182-188. doi:10.1111/his.13975.

[18] Jun JK, Choi KS, Lee H-Y, Suh M, Park B, Song SH, et al. Effectiveness of the Korean NCSP in reducing gastric cancer mortality. Gastroenterology. 2017;152:1319-1328.e7. doi:10.1053/j.gastro.2017.01.029.

[19] Park CH, Kim EH, Jung DH, Kim JH, Lee H, et al. Operational definitions for H. pylori infection using claims data in Korea. J Korean Med Sci. 2023;38:e169. doi:10.3346/jkms.2023.38.e169.

**Supplementary Table S2 -** External validation dataset: cancer screenee cohort

| **Characteristics** | **Total**  **(n=31,754)** | **No gastric cancer**  **(n=31,254)** | **Gastric cancer**  **(n=31)** | ***P*-value** |
| --- | --- | --- | --- | --- |
| **Age** | | | | 0.007 |
| 40–44 |  | 6,150 (19.7) | 3 (9.7) |  |
| 45–49 |  | 7,297 (23.3) | 4 (12.9) |  |
| 50–54 |  | 6,902 (22.1) | 6 (19.4) |  |
| 55–59 |  | 4,989 (15.7) | 6 (19.4) |  |
| 60–64 |  | 3,524 (11.3) | 4 (12.9) |  |
| ≥65 |  | 2,483 (7.9) | 8 (25.8) |  |
| **Sex** | | | | 0.092 |
| Male |  | 15,940 (51.0) | 21 (67.7) |  |
| Female |  | 15,314 (49.0) | 10 (32.3) |  |
| **BMI (kg/m^2^)** | | | | 0.134 |
| <23 |  | 11,740 (37.6) | 7 (22.6) |  |
| 23–24.9 |  | 8,808 (28.2) | 13 (41.9) |  |
| 25–29.9 |  | 9,857 (31.5) | 9 (29.0) |  |
| ≥30 |  | 849 (2.7) | 2 (6.5) |  |
| **Smoking** | | | | 0.014 |
| Non-Smoker |  | 17,508 (56.0) | 13 (41.9) |  |
| Ex-Smoker |  | 6,751 (21.6) | 17 (54.8) |  |
| Smoker |  | 6,995 (22.4) | 1 (3.2) |  |
| **Drinking** | | | | 0.977 |
| No drinking |  | 13,479 (43.1) | 13 (41.9) |  |
| ≤ 3 times/week |  | 16,935 (54.2) | 17 (54.8) |  |
| ≥ 4 times/week |  | 840 (2.7) | 1 (3.2) |  |
| **Family history of gastric cancer** | | | | 0.019 |
| No |  | 29,638 (94.8) | 26 (83.9) |  |
| Yes |  | 1,616 (5.2) | 5 (16.1) |  |
| **Family history of colorectal cancer** | | | | 0.998 |
| No |  | 30,583 (97.9) | 30 (96.8) |  |
| Yes |  | 671 (2.1) | 1 (3.2) |  |
| **Family history of liver cancer** | | | | 0.941 |
| No |  | 30,392 (97.2) | 30 (96.8) |  |
| Yes |  | 862 (2.8) | 1 (3.2) |  |
| **Hypertension** | | | | 0.938 |
| No |  | 25,541 (81.7) | 27 (87.1) |  |
| Yes |  | 5,713 (18.3) | 4 (12.9) |  |
| **Diabetes** | | | | 0.188 |
| No |  | 30,520 (97.7) | 29 (93.5) |  |
| Yes |  | 734 (2.3) | 2 (6.5) |  |
| **Hyperlipidemia** | | | | 0.787 |
| No |  | 31,169 (99.7) | 29 (93.5) |  |
| Yes |  | 85 (0.3) | 2 (6.5) |  |
| **Myocardial infarction or angina pectoris** | | | | 0.932 |
| No |  | 31,157 (99.7) | 26 (83.9) |  |
| Yes |  | 97 (0.3) | 5 (16.1) |  |
| **Stroke** | | | | 0.669 |
| No |  | 30,180 (96.6) | 26 (83.9) |  |
| Yes |  | 1,074 (3.4) | 5 (16.1) |  |
| **Colorectal cancer** | | | | 0.031 |
| No |  | 31,094 (99.5) | 29 (93.5) |  |
| Yes |  | 160 (0.5) | 2 (6.5) |  |
| **Liver cancer** | | | | 0.013 |
| No |  | 31,202 (99.8) | 29 (93.5) |  |
| Yes |  | 52 (0.2) | 2 (6.5) |  |
| ***Helicobacter pylori* infection** | | | | 0.014 |
| No |  | 20,248 (64.8) | 15 (48.4) |  |
| Yes |  | 11,006 (35.2) | 16 (51.6) |  |
| **Atrophic gastritis or intestinal metaplasia** | | | | 0.011 |
| No |  | 24,534 (78.5) | 18 (58.1) |  |
| Yes |  | 6,720 (21.5) | 13 (41.9) |  |
| **Gastric adenoma** | | | | 0.002 |
| No |  | 31,162 (99.7) | 29 (93.5) |  |
| Yes |  | 92 (0.3) | 2 (6.5) |  |
